# Nonequivalent modulation of corticospinal excitability by positive and negative outcomes

**DOI:** 10.1002/brb3.862

**Published:** 2017-12-12

**Authors:** Makoto Suzuki, Toyohiro Hamaguchi, Atsuhiko Matsunaga

**Affiliations:** ^1^ School of Health Sciences Saitama Prefectural University Saitama Japan; ^2^ School of Allied Health Sciences Kitasato University Kanagawa Japan

**Keywords:** behavior, corticospinal excitability, magnetic stimulation, reward

## Abstract

**Objective:**

The difference between positive and negative outcomes is important in trial‐and‐error decision‐making processes and affects corticospinal excitability. This study investigated corticospinal excitability during the performance of trial‐and‐error decision‐making tasks with varying competing behavioral outcomes.

**Methods:**

Each trial began with one of five colored circles presented as a cue. Each color represented a different reward probability, ranging from 10% to 90%. The subjects were instructed to decide whether to perform wrist flexion in response to the cue. Two seconds after the presentation of the cue, a reward stimulus (picture of a coin) or penalty stimulus (mauve circle) was randomly presented to the subject. If the picture of a coin appeared, the subjects received the coin after the experiment if they had performed wrist flexion, but not if they had not performed wrist flexion. If a mauve circle appeared, a coin was deducted from the total reward if the subjects had performed wrist flexion, but not if they had not performed wrist flexion. One second after the reward or penalty stimulus, transcranial magnetic stimulation was delivered to the primary motor cortex at the midpoint between the centers of gravity of the flexor carpi radialis (agonist) and extensor carpi radialis (antagonist) muscles.

**Results:**

Cumulative wrist flexions were positively correlated with reward probabilities. Motor evoked potential (MEP) amplitudes in agonist muscles were significantly higher when wrist flexion incurred a penalty than when it incurred a reward, but there was no difference in the MEP amplitudes of antagonist muscles.

**Conclusion:**

Positive and negative behavioral outcomes differentially altered behavior and corticospinal excitability, and unexpected penalties had a stronger effect on corticospinal excitability for agonist muscles.

## 1 INTRODUCTION

In everyday life, people usually learn from positive and negative behavioral outcomes to reinforce rewarded behaviors and avoid nonrewarded behaviors (Herzfeld, Vaswani, Marko, & Shadmehr, 2014; Klein et al., 2007). Behavioral learning is often described as a trial‐and‐error decision‐making process involving interacting social, economic, psychological, and neurophysiological aspects (Cushman, Murray, Gordon‐McKeon, Wharton, & Greene, 2012; Fleming, Thomas, & Dolan, 2010; Nicolle, Fleming, Bach, Driver, & Dolan, 2011; Pisoni et al., 2014; Sanfey, 2007). When one must make difficult choices, decisions are based on competing positive and negative outcomes and are influenced by one's history of choices and the contexts under which the choices were made (Akaishi, Umeda, Nagase, & Sakai, 2014; Galea, Ruge, Buijink, Bestmann, & Rothwell, 2013). For example, if one expects mostly positive outcomes, then there will be more negative prediction errors in which outcomes fail to meet expectations and fewer positive prediction errors in which outcomes exceed expectations. The number of prediction errors is therefore related to discrepancies between expectations and outcomes. Positive and negative prediction errors both induce synaptic plasticity in cortical and subcortical structures including the primary motor cortex (M1), leading to reinforcement of existing behaviors or selection of new behaviors (Calabresi, Picconi, Tozzi, & Di Filippo, 2007; Centonze et al., 2003; Davis, Coyne, & McNeill, 2007; Lang et al., 2008; Molina‐Luna et al., 2009; Wickens, Reynolds, & Hyland, 2003). Therefore, the impact of positive and negative outcomes has been explored at the corticospinal and behavioral levels.

Positive and negative behavioral outcomes can reinforce approach and avoidance behaviors, respectively (Coombes, Janelle, & Duley, 2005; Naugle, Joyner, Hass, & Janelle, 2010). Transcranial magnetic stimulation (TMS) can be used to probe the effects of approach and avoidance behaviors on corticospinal excitability because TMS activates the corticospinal system and elicits motor evoked potentials (MEPs) that reflect behavioral outcome‐induced changes in corticospinal excitability (Kapogiannis, Campion, Grafman, & Wassermann, 2008; Thabit et al., 2011). Desirable outcomes, such as monetary rewards, increase MEP amplitudes in agonist muscles for the rewarded action (Borgomaneri, Gazzola, & Avenanti, 2014; Gupta & Aron, 2011; Kapogiannis et al., 2008, 2011; Thabit et al., 2011), but viewing upsetting images also increases MEP amplitudes (Borgomaneri, Gazzola, & Avenanti, 2012; Coelho, Lipp, Marinovic, Wallis, & Riek, 2010; Oliveri et al., 2003). These observations suggest that positive and negative signals modulate M1 motor output and MEPs in agonist muscles. However, because these studies used observational settings (Borgomaneri et al., 2012; Coelho et al., 2010; Kapogiannis et al., 2008; Oliveri et al., 2003; Pisoni et al., 2014) or experimental tasks with predetermined reward probabilities without a specified movement (Borgomaneri et al., 2014; Suzuki et al., 2014; Thabit et al., 2011), it is impossible to know whether the observed outcome‐related corticospinal excitability changes were specific to selected muscle movement behaviors. In particular, even if the subjects performed the task successfully, the behavioral results did not reflect the predetermined reward probabilities. Therefore, although muscle‐related corticospinal excitability changes are associated with behavioral outcome expectations, researchers do not yet understand their relationship with gain or loss probabilities or whether discrepancies between expectations and outcomes affect M1 excitability and behavior selection. These are serious lacunae, since such data could potentially elucidate the relationship between muscle movement behaviors and MEP amplitude changes in active muscles during behavioral learning in the context of competing positive and negative behavioral outcomes. In addition to expanding on previous findings, exploring how positive and negative outcomes affect behavioral choices and corticospinal excitability may have interesting implications in behavior analysis and neuroscience.

**Figure 1:**
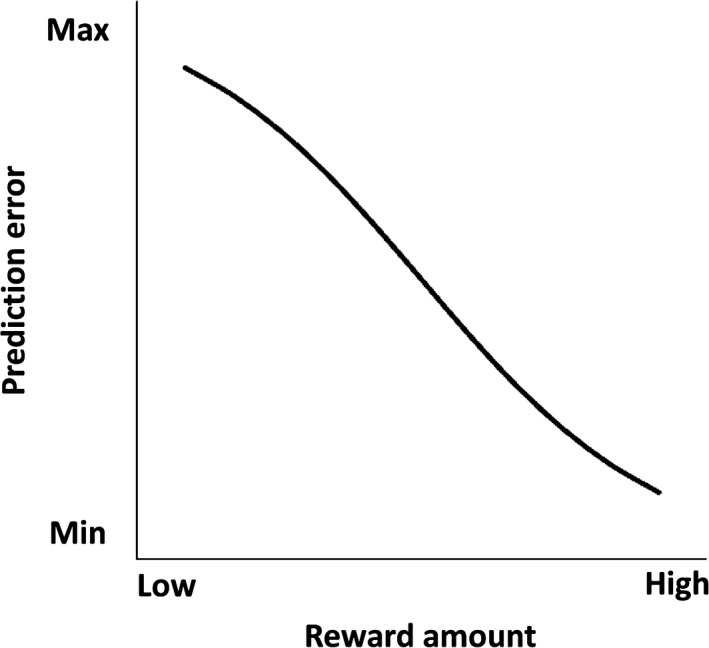
Hypothesized relationship between prediction errors and reward numbers. Negative prediction errors become maximal when unexpected penalties are frequently incurred and expected rewards rarely are, whereas positive prediction errors become minimal when expected rewards are frequently earned and unexpected penalties rarely are

Because the temporal resolution of TMS is adequate for observing corticospinal excitability changes induced by behavioral outcomes, we designed a paradigm involving varying probabilities of positive and negative outcomes for behavioral choices. This paradigm facilitates the investigation of both M1 excitability and behavior selection in the context of trial‐and‐error decision‐making with competing outcomes. In a task where performing an action could incur a reward or a penalty, there were few positive prediction errors despite the number of rewards being large. However, there were many negative prediction errors despite the number of rewards being small (Figure 1). This raises the question of whether corticospinal excitability reflects prediction errors or the number of rewards. We predicted that if prediction errors affect M1 excitability, then unexpected penalties should increase MEP amplitudes more than expected rewards do. We therefore used TMS to investigate corticospinal excitation during trial‐and‐error decision‐making tasks with competing behavioral outcomes.

## 2 METHOD

### 2.1 Participants

We based our target sample size on a desired 90% statistical power to detect changes in behaviors and peak‐to‐peak MEP amplitudes with a 0.90 effect size and a two‐sided α‐level of 0.05. Inputting these parameters into the Hulley matrix (Hulley & Cummings, 1988) yielded a sample size of 12. Accordingly, we recruited 13 healthy, neurologically intact subjects (six men and seven women aged 20–29 years, mean ± standard deviation [*SD*]: 21.9 ± 2.3 years) for the behavioral and MEP amplitude measurements. The subjects did not know the experiment's purpose, and screening showed that none was at risk of adverse events from TMS (Wassermann, 1998). None took medications or had any psychiatric or neurological diseases. We confirmed right‐handedness with the Edinburgh Handedness Inventory (Oldfield, 1971), recording a mean laterality quotient score of 0.9 points (*SD*: 0.2 points). The experimental procedures were approved by the Research Ethics Committee of the Kitasato University School of Allied Health Sciences and performed in accordance with the principles of the Declaration of Helsinki. All subjects provided written informed consent prior to participation.

### 2.2 Experimental setup

Each subject sat comfortably in front of a 25.7‐cm screen located 50 cm from the face at eye level with the right palm and forearm resting on the test equipment (Support Jig, Kyoei Engineering, Niigata, Japan). The right forearm was fixed to a particle‐foam plastic support cushion, and the right hand was placed in a hand‐piece that strapped the fingers in a flexed position. The wrist could freely flex, and after wrist flexion, the equipment automatically returned the wrist to the neutral starting position.

### 2.3 Electromyographic (EMG) recordings

The motor output from any given cortical site elicits movement, with signals with different “gains” converging on multiple muscles (Huntley & Jones, 1991; Melgari, Pasqualetti, Pauri, & Rossini, 2008). Agonist–antagonist coordination in particular is necessary to execute quick movements (Gottlieb, 1998; Pfann, Buchman, Comella, & Corcos, 2001). Several surface EMG studies have reported a significant increase in agonist activation during behaviors (Geertsen, Lundbye‐Jensen, & Nielsen, 2008; Suetta et al., 2004). Some found a decrease in antagonist coactivation (Carolan & Cafarelli, 1992), but others did not (Aagaard et al., 2000). We therefore recorded surface EMG activity from the flexor carpi radialis (FCR) and extensor carpi radialis (ECR) muscles using disposable, self‐adhesive Ag‐AgCl electrodes (M‐00‐S; Mets, Tokyo, Japan) to assess the corticospinal excitability changes divergently affecting the agonist FCR and antagonist ECR muscles during behavioral tasks. The electrode centers were located 2 cm apart over the thickest portion of the muscle and longitudinally aligned with the muscle fiber direction (Bertolasi, Priori, Tinazzi, Bertasi, & Rothwell, 1998; Rota, Morel, Saboul, Rogowski, & Hautier, 2014; Stowe, Hughes‐Zahner, Stylianou, Schindler‐Ivens, & Quaney, 2008). The EMG signals were amplified ×100, bandpass filtered at 5–2,000 Hz with a DL‐140 amplifier (4Assist, Tokyo, Japan), digitized at 10 kHz with a PowerLab system (ADInstruments, Dunedin, New Zealand), and stored on magnetic media.

### 2.4 Transcranial magnetic stimulation

Transcranial magnetic stimulation was delivered to the scalp through a figure‐eight coil (internal diameter of each wing: 70 mm) using a Magstim 200^2^ stimulator (Magstim, Whitland, UK) (Rossini, Rossini, & Ferreri, 2010). To induce a current from the posterolateral to anteromedial left brain, the coil was held tangentially to the scalp at approximately 45° to the midline, and the handle was pointed dorsolaterally. At the experiment's start, we found the optimal coil position for eliciting maximal MEPs in each FCR and ECR (the so‐called “hot spot”) by moving the coil over the left M1 (Mang, Clair, & Collins, 2011) and then marked it with a soft‐tipped pen. The hot spot's resting motor threshold (RMT) was defined as the minimum stimulus intensity required to elicit an MEP in the relaxed FCR or ECR of at least 50 μV in 5 out of 10 consecutive trials. The stimulus intensity started at 40% of maximum stimulator output and was then altered in 1% increments and decrements.

### 2.5 Motor representational map

To map muscle representations, a 6 × 6 cm^2^ grid of 25 positions with 1.5‐cm spacings was marked on each subject's head, and its center was on the hot spot described in subsection 2.4 (Suzuki et al., 2014). For each scalp position, we recorded the MEPs evoked by five stimuli at 120% of the RMT delivered in a clockwise spiral beginning at the hot spot (interstimulus interval: 5 s). The center of gravity (CoG) was computed separately for each muscle as a measure of the amplitude‐weighted center on a motor representational map with reference to Cz in the international 10–20 system (Marconi et al., 2011; Meesen, Cuypers, Rothwell, Swinnen, & Levin, 2011; Suzuki et al., 2012). This was expressed as a bivariate measurement with anteroposterior (*x*) and mediolateral (*y*) coordinates according to the following formula:

$$\text{CoG}=\left[\frac{\sum a_{i}x_{i}}{\sum a_{i}},\frac{\sum a_{i}y_{i}}{\sum a_{i}}\right],$$

where *x*_*i*_ and *y*_*i*_ are the stimulus coordinates, and *a*_*i*_ is the MEP amplitude. CoGs correspond to the locations of the most excitable neuron populations projecting to the target muscle (Devanne, Lavoie, & Capaday, 1997; Rosenkranz, Kacar, & Rothwell, 2007; Smyth, Summers, & Garry, 2010; Suzuki et al., 2012).

### 2.6 Behavioral task

**Figure 2:**
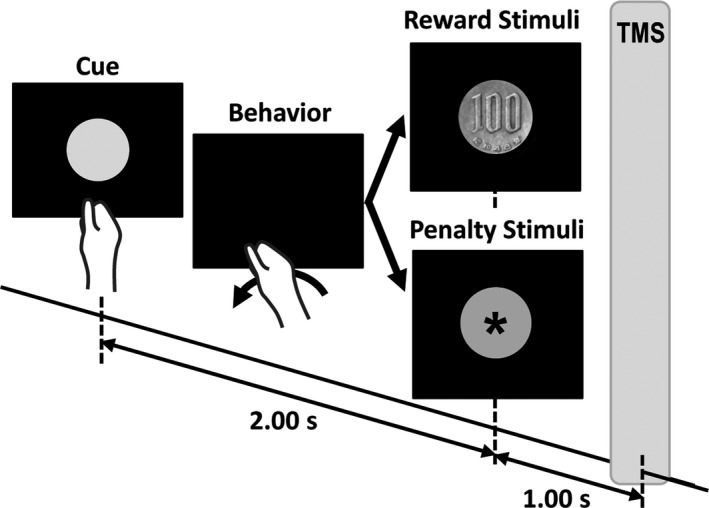
Experimental design for the trial‐and‐error decision‐making task. The subject sat in front of a black screen. Each trial began with one of five colored circles presented as a cue. Each color represented a different reward probability, ranging from 10% to 90%. The subject was instructed to decide whether or not to perform wrist flexion in response to the cue. Two seconds after the presentation of the cue, a reward stimulus (a picture of a coin) or a penalty stimulus (a mauve circle) was presented to the subject. If the picture of a coin appeared, the subject received the coin after the experiment if they had performed wrist flexion, but not if they had not performed wrist flexion. If a mauve circle appeared, a 100‐yen coin was deducted from the total reward if the subject had performed wrist flexion, but not if they had not performed wrist flexion. Single‐pulse TMS was delivered 1 s after the appearance of the reward or penalty stimulus. The intertrial interval was 4 s. TMS, transcranial magnetic stimulation

Each trial began with a 2‐s cue consisting of a random presentation of one of five colored circles (blue, red, yellow, black, or green) that represented different reward probabilities (.9, .7, .5, .3, and .1, respectively) (Figure 2). The subjects were told that each color represented a different reward probability, but they were not told which probability each color represented. A reward probability of .9 meant that rewards (Thabit et al., 2011) and penalties would be presented in 90% and 10% of trials, respectively. Thus, each color represented both reward and penalty probabilities. The subject had to decide as quickly as possible whether to perform wrist flexion in response to the cue. We recorded the number of flexion events for each color cue. Two seconds after the cue presentation, a reward stimulus (a picture of a ¥100 coin) or a penalty stimulus (a mauve circle containing an asterisk) (Thabit et al., 2011) was presented for 2 s as feedback. If wrist flexion had occurred during the cue presentation, then a reward or penalty stimulus indicated that the subject would receive or lose, respectively, ¥100 after the experiment. Nonflexion caused no reward or penalty. Each subject received the net earned monetary reward after the experiment. The subjects had to learn to perform wrist flexion for the colors representing high reward probabilities and not perform wrist flexion for those representing low reward probabilities through trial‐and‐error decision‐making. Under this paradigm, reward anticipation increases when subjects perform wrist flexion for colors that they believe have high reward probabilities based on their experiences. However, reward anticipation decreases when subjects avoid wrist flexion for colors that they believe have low reward probabilities. There would therefore be few positive prediction errors despite the number of rewards being large, but many negative prediction errors despite the number of rewards being small.

This trial‐and‐error decision‐making task comprised 100 trials, and each color was presented 20 times. The intertrial interval was 4 s. One second after the presentation of the reward or penalty stimulus, we delivered a single‐pulse TMS of 120% of the FCR's RMT at the midpoint between the CoGs of the FCR and ECR (Gupta & Aron, 2011; Kapogiannis et al., 2008; Suzuki et al., 2014; Thabit et al., 2011). Schultz (2007) noted that dopamine concentrations peak approximately 1 s after the onset of the reward‐related stimulus, start declining after 2 s, and return to baseline after approximately 4 s. Borgomaneri et al. (2012) noted that M1 excitability changed at least 300 ms after the presentation of negative emotional pictures. Thabit et al. (2011) detected M1 excitability changes occurring 1 s after monetary reward or nonreward stimuli presented at 3‐ to 4‐s intervals. We considered these time courses when choosing the TMS delivery time and intertrial interval in our protocol.

### 2.7 Data analysis

During the experiment, we quantified behaviors for each color cue stimulus as the cumulative number of wrist flexion events and the ratio of that number to the total number of stimulus presentations. This ratio was quantified throughout the task in 10‐trial bins and analyzed with a time series analysis conducted by calculating the autocorrelation function with a 1‐bin time lag. The behavioral pattern time courses for color cues with opposite reward and loss probabilities were identified using *R* ^2^ values (Gao, Zheng, & Wang, 2010; Nelson‐Wong, Howarth, Winter, & Callaghan, 2009; Roy Choudhury et al., 2011; Schmidt et al., 2010). Each subject's wrist flexion and MEP data were then normalized by linear transformation, and the data were expressed as Z scores (Aglioti, Cesari, Romani, & Urgesi, 2008) because they were nonnormally distributed. We compared flexion and MEP data across reward probabilities using Friedman's test and post hoc analysis with the Steel‐Dwass test. These analyses revealed the relative changes in behavior and prediction errors based on positive and negative outcomes.

Motor evoked potential amplitudes for trials with or without wrist flexion were compared with the Mann–Whitney *U* test. We also compared MEP amplitudes across trials in which flexion earned a reward, nonflexion forfeited a reward, flexion incurred a penalty, or nonflexion avoided a penalty. These analyses revealed the relative changes in corticospinal excitability and prediction errors based on positive and negative outcomes. All data are expressed as mean ± standard error of the mean or medians and interquartile ranges. We defined statistical significance as *p *< .05. All statistical tests were performed with PASW Statistics 18 software (IBM, Armonk, NY) and R 3.3.0 software (R Foundation for Statistical Computing, Vienna, Austria).

## 3 RESULTS

All subjects completed all experimental conditions. No adverse TMS‐related effects occurred during the experiments.

### 3.1 Motor representational map

The RMTs of the FCR and ECR were 48.6% ± 1.6% and 45.6% ± 1.6%, respectively, of the maximum stimulator output. The reciprocal muscle areas clearly overlapped, but they were not identical. The CoGs for the FCR and ECR were located at (7.4 ± 2.3 mm, 56.6 ± 1.9 mm) and (4.1 ± 3.1 mm, 57.8 ± 2.1 mm), respectively. The midpoint between the two CoGs was located at (5.7 ± 2.6 mm, 57.2 ± 1.9 mm), and the coil was placed there.

### 3.2 Flexion results

**Figure 3:**
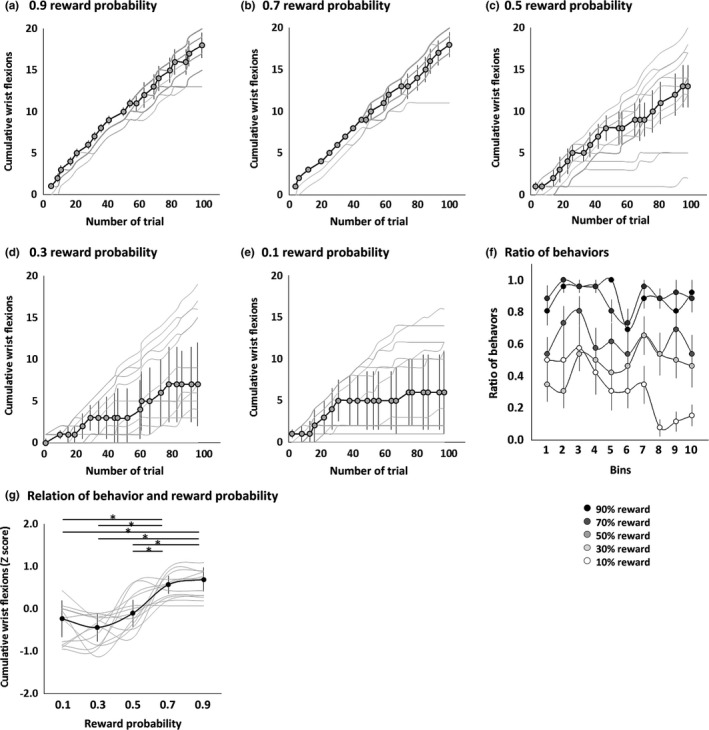
Behaviors for .9 (a), .7 (b), .5 (c), .3 (d), and .1 (e) reward probabilities. Gray lines denote each subject result. The symbols and error bars denote median and interquartile range. The cumulative wrist flexions increased over time for all reward probabilities although intersubject variability increased with lower reward probabilities. The proportion of trials featuring wrist flexion (f) for each color both throughout the task and in 10‐trial bins remained almost stable with random fluctuations after the first bin, except for the .1 reward probability (f). The cumulative wrist flexions at 0.9 and 0.7 reward probabilities were significantly increased than those at lower probabilities (*p < .05) (g)

The numbers of wrist flexion events for the 0.9, 0.7, 0.5, 0.3, and 0.1 reward probabilities were 17.8 ± 0.6, 18.0 ± 0.7, 12.5 ± 1.5, 9.5 ± 1.7, and 7.1 ± 1.6, respectively. Figure 3 shows that the cumulative wrist flexions increased over time for all reward probabilities, although intersubject variability increased with lower reward probabilities. The proportion of trials in which the subject performed wrist flexion remained almost fixed after the first bin and fluctuated randomly for reward probabilities from .3 to .9 (Figure 3f). Most autocorrelation values with a 1‐bin time lag were nonsignificant (.9 reward probability: *R* ^2 ^= 0.018, *p *=* *.730; .7 reward probability: *R* ^2 ^= 0.011, *p *=* *.788; .5 reward probability: *R* ^2 ^= 0.038, *p *=* *.616; and .3 reward probability: *R* ^2 ^= 0.078, *p *=* *.466). For the .1 reward probability, the proportion of trials involving wrist flexion gradually decreased over the task (*R* ^2 ^= 0.600, *p *=* *.014). The different reward probabilities were associated with significantly different cumulative wrist flexions (Friedman's test, *p *<* *.0001; Figure 3g). The post hoc Steel‐Dwass tests showed that there were significantly more cumulative wrist flexions at the .9 and .7 reward probabilities than at lower reward probabilities (Table 1).

**Table 1:** Cumulative wrist flexions between reward probabilities

Reward probability	.9	.7	.5	.3	.1
.9	–				
.7	.963	–			
.5	.009^a^	.021^a^	–		
.3	.0001^a^	.0001^a^	.106	–	
.1	.0006^a^	.0006^a^	.470	.831	–

a *p *<* *.05.

### 3.3 Corticospinal excitability

**Figure 4:**
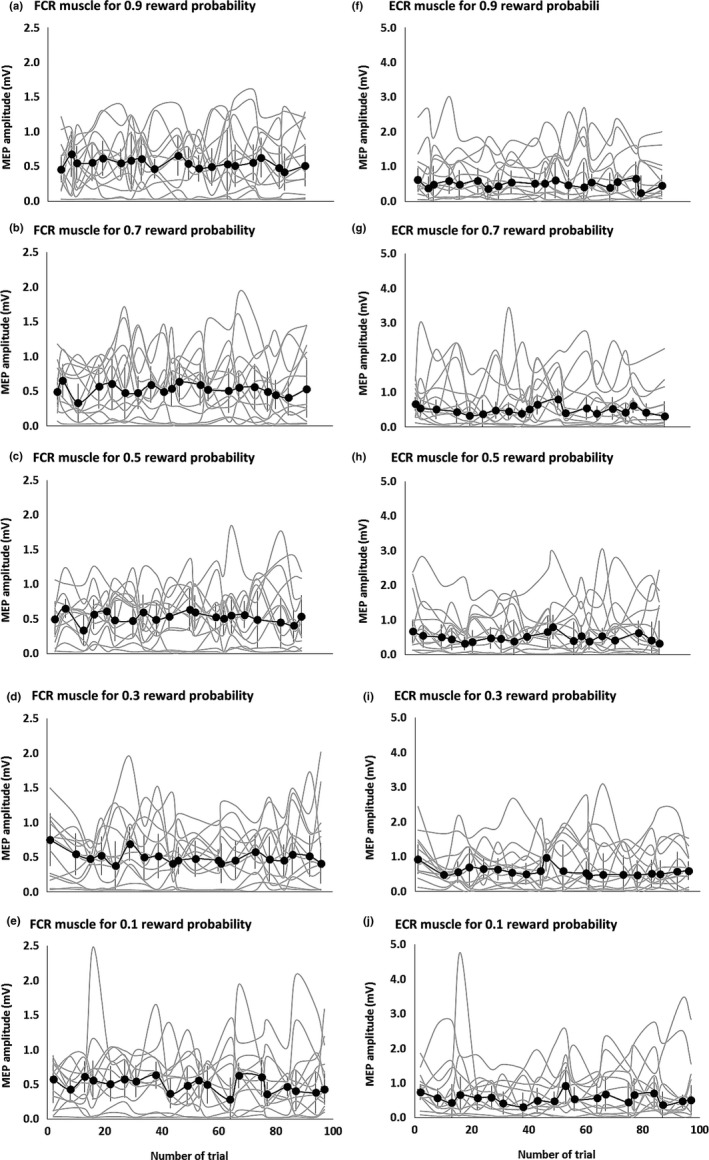
The MEP amplitudes of FCR (left) and ECR (right) muscles during the trial‐and‐error decision‐making tasks. The symbols and error bars denote median and interquartile range. The MEP amplitudes of the FCR for 0.9 (a), 0.7 (b), 0.5 (c), 0.3 (d), and 0.1 (e) reward probabilities and ECR muscles for 0.9 (f), 0.7 (g), 0.5 (h), 0.3 (i), and 0.1 (j) reward probabilities. MEP amplitude changes across trials were jumbled and small, with the values being generally stable. FCR, flexor carpi radialis; ECR, extensor carpi radialis; MEP, motor evoked potential

**Figure 5:**
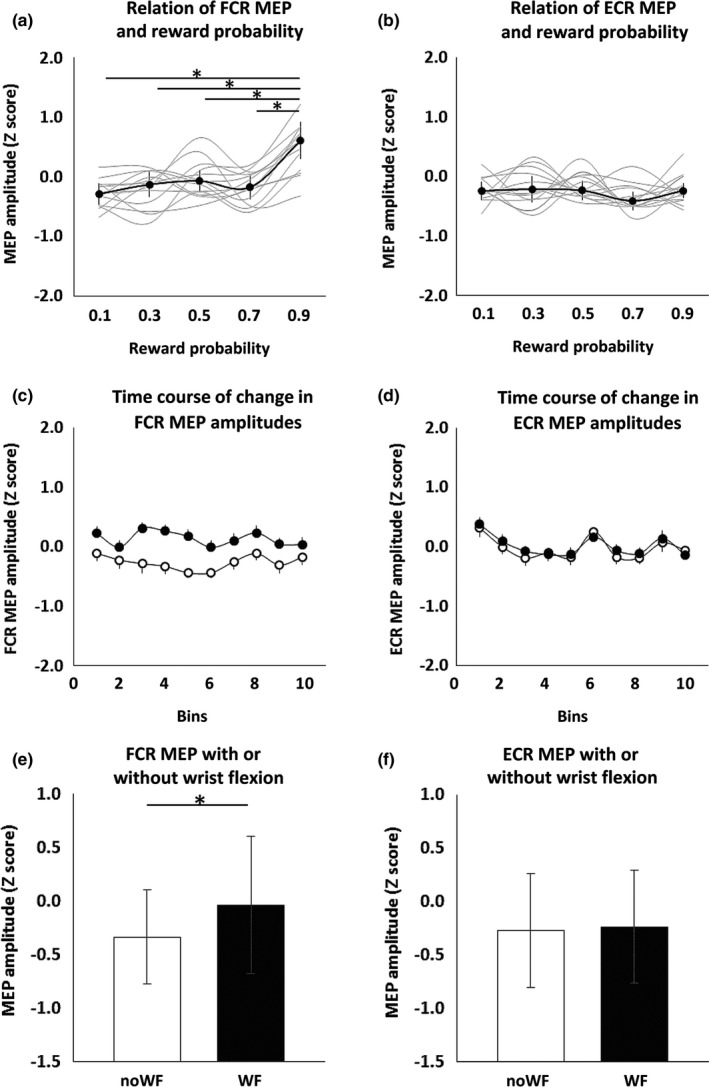
The MEP amplitudes of FCR (left) and ECR (right) muscles with and without wrist flexion. The symbols and error bars denote median and interquartile range. The MEP amplitude of agonist FCR muscle (a) for .9 reward probability was significantly higher than those for .7, .5, .3, and .1 reward probabilities (*p <* *.05). However, there were no significant differences between antagonist ECR MEP amplitude and reward stimuli presentations (b). Time course of changes in FCR (c) and ECR (d) MEP amplitudes with (filled symbols) and without (open symbols) wrist flexion. The time courses were generally stable throughout the task. The MEP amplitude of FCR muscle with wrist flexion was significantly higher than without wrist flexion (e) (*p <* *.05), whereas that of the ECR muscle was not significantly different (f) (*p *=* *.970). FCR, flexor carpi radialis; ECR, extensor carpi radialis; MEP, motor evoked potential; noWF, without wrist flexion; WF, with wrist flexion

Figure 4 shows the MEP amplitudes of the FCR and ECR during the trial‐and‐error decision‐making tasks. MEP amplitude changes across trials were jumbled and small, with the values being generally stable. The FCR MEP amplitudes were significantly different between reward probabilities (Friedman's test, *p *<* *.0001; Figure 5a), whereas those of ECR were not (*p *=* *.587; Figure 5b). The post hoc Steel‐Dwass tests showed that the FCR MEP amplitudes were significantly greater at the .9 reward probability than at the other reward probabilities (Table 2).

**Table 2:** Peak‐to‐peak MEP amplitudes obtained for the FCR muscle between reward probabilities

Reward probability	.9	.7	.5	.3	.1
.9	–				
.7	.006^a^	–			
.5	.031^a^	.946	–		
.3	.004^a^	.997	.878	–	
.1	.001^a^	.805	.254	.878	–

a *p *<* *.05.

FCR, flexor carpi radialis; MEP, motor evoked potential.

**Figure 6:**
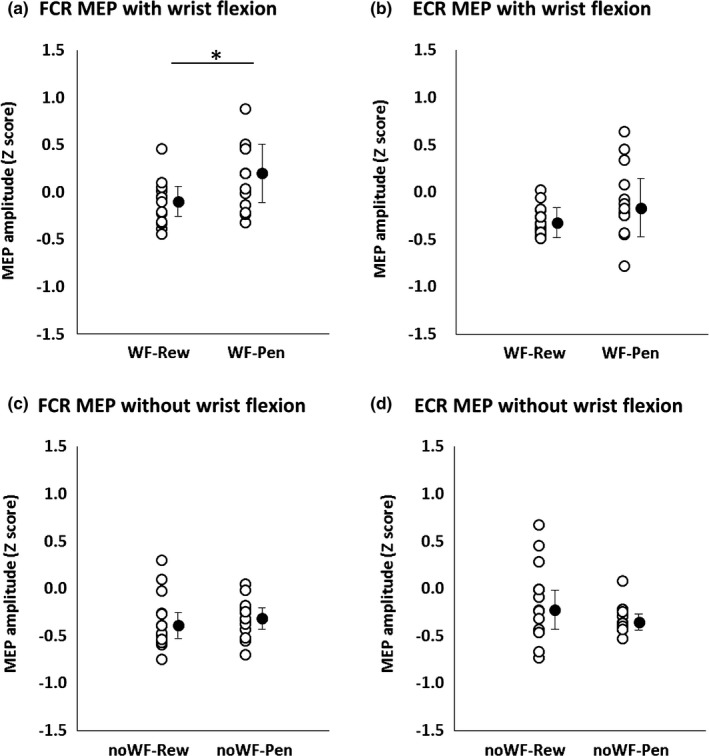
The relation between MEP amplitude and positive and negative behavioral outcomes. The gray circle denote each subject's median data. The black circles and error bars denote median and interquartile range. FCR MEP amplitude with a penalty stimulus and wrist flexion was significantly higher than that with a reward stimulus and wrist flexion (*p =* *.016) (a). However, ECR MEP amplitude with wrist flexion (*p *=* *.196) (b), FCR MEP amplitude without wrist flexion (*p *=* *.972) (c) and ECR MEP amplitude without wrist flexion (*p *=* *.272) (d) were not significantly different. FCR, flexor carpi radialis; ECR, extensor carpi radialis; MEP, motor evoked potential; noWF, without wrist flexion; WF, with wrist flexion; Rew, with reward; noRew, without reward

Figure 5c,d shows the time courses of changes in FCR and ECR MEP amplitudes with and without wrist flexion. Mean MEP amplitudes in 10‐trial bins were generally stable. However, FCR MEP amplitudes were significantly greater with wrist flexion than without it (Mann–Whitney *U* test, *p *<* *.0001) (Figure 5e), but no such difference was observed for the ECR (*p *=* *.970) (Figure 5f). Additionally, FCR MEP amplitudes were significantly higher when wrist flexion incurred a penalty than when it earned a reward (*p *=* *.016) (Figure 6a). However, MEP amplitudes were not significantly different between any other stimulus‐behavior conditional pairs (Figure 6b–d).

## 4 DISCUSSION

To test the hypothesis that nonequivalent positive and negative behavioral outcomes should produce unequal MEP amplitudes, we measured changes in behavior and corticospinal excitability related to positive and negative outcomes during a trial‐and‐error decision‐making task. Our results showed that (a) cumulative wrist flexions increased with greater reward probabilities; (b) behaviors fluctuated randomly for most reward probabilities; (c) agonist FCR MEP amplitudes were increased in trials where wrist flexion incurred a penalty, but antagonist ECR MEP amplitudes were not; and (d) MEP amplitudes in the agonist and antagonist muscles did not change in trials without wrist flexion regardless of the outcome. These observations show that outcome histories modulated behavior and that unexpected penalties affected corticospinal excitation of the agonist muscle. In fact, FCR MEP amplitudes were significantly higher when wrist flexion incurred a penalty than when it earned a reward. This is surprising and implies that positive and negative outcomes do not equally affect MEP amplitude changes in agonist muscles. Furthermore, agonist muscle MEP amplitudes are affected by negative prediction errors rather than by the number of expected rewards. This is the first systematic study to show that unexpected outcomes change behavior and corticospinal excitability during trial‐and‐error decision‐making.

In decision‐making, expected outcomes are evaluated based on whether they improve or worsen the decision‐maker's current condition (Cos, Duque, & Cisek, 2014; Fee & Goldberg, 2011; Fleming et al., 2010; Galea et al., 2013; Herzfeld et al., 2014; Nicolle et al., 2011; Pisoni et al., 2014; Rangel, Camerer, & Montague, 2008; Samejima & Doya, 2007). The concepts of reward and penalty have been used to show that dopamine‐dependent processes select behaviors that maximize rewards and minimize penalties (Berns, McClure, Pagnoni, & Montague, 2001; Frank, Seeberger, & O'Reilly, 2004; Galea et al., 2013; McClure, Berns, & Montague, 2003; O'Doherty, Critchley, Deichmann, & Dolan, 2003; Pessiglione, Seymour, Flandin, Dolan, & Frith, 2006; Schultz, Dayan, & Montague, 1997; Tanaka et al., 2006). In our study, we observed that more wrist flexions occurred at higher reward probabilities and that this could be explained as learning from unexpected penalties. Furthermore, the proportion of trials featuring wrist flexion was almost constant after the first 10 trials, and these behaviors fluctuated randomly for reward probabilities from .3 to .9. This stable change with random fluctuations suggests that the subjects distinguished colors representing high reward probabilities from those representing low reward probabilities in the first 10 trials and that their discrimination varied between the status quo and random new behaviors in later trials. These results provide new evidence that behavioral selection changes reflect unexpected penalties rather than expected rewards.

Previous studies (Kapogiannis et al., 2008; Thabit et al., 2011) suggested that corticospinal excitability changes are associated with reward expectations and are modified by prior experience. Additionally, highly desirable stimuli increase corticospinal excitability more than less desirable or neutral stimuli (Gupta & Aron, 2011). Recently, Suzuki et al. (2014) reported that agonist MEP amplitudes are greater than antagonist MEP amplitudes after reward stimuli, with larger differences for higher reward probabilities, but not after neutral stimuli. In contrast, negative outcomes can also elicit corticospinal excitability. Previous studies (Borgomaneri et al., 2012; Coelho et al., 2010; Oliveri et al., 2003) noted that MEP amplitudes are greater during negative stimuli than during neutral stimuli or rest. These findings suggest that cortical motor outputs are modulated by reward and penalty signals. However, we found that agonist FCR MEP amplitudes were increased by penalties rather than rewards. This finding is markedly inconsistent with the findings of previously published studies using positive stimuli (Borgomaneri et al., 2014; Gupta & Aron, 2011; Suzuki et al., 2014; Thabit et al., 2011). The subjects in these studies were either resting motionlessly without the need to move (Borgomaneri et al., 2012; Kapogiannis et al., 2008; Oliveri et al., 2003) or their behaviors did not reflect the predetermined reward probabilities (Borgomaneri et al., 2014; Gupta & Aron, 2011; Suzuki et al., 2014; Thabit et al., 2011), so it is impossible to tell whether the observed outcome‐related corticospinal excitability changes were specific to behavioral choices. In contrast, our task involved behavioral choices with the risk of penalties. Our subjects expected rewards when they performed wrist flexion, and they made few positive prediction errors but many negative prediction errors. This design allowed us to investigate whether corticospinal excitability reflected prediction errors or the number of rewards in the context of muscle movement behaviors. Our results suggested that cortical motor outputs are more strongly modulated by unexpected penalties than by expected rewards. Thus, our results expand upon those of previous studies that used negative stimuli in observational settings (Borgomaneri et al., 2012; Coelho et al., 2010; Oliveri et al., 2003).

Our paradigm was designed to manipulate reward and penalty probabilities, and we predicted that unexpected outcomes would dominate the modulation of corticospinal excitability. Our primary aim was to test whether positive or negative outcomes affect corticospinal excitability and behavior selection. We found that muscle activation affected agonist MEP amplitudes because the agonist FCR's MEP amplitudes were higher with wrist flexion than without it. We therefore compared MEP amplitudes with or without wrist flexion and with reward or penalty stimuli and found that the agonist FCR's MEP amplitudes were increased with wrist flexion following penalty stimuli, but the antagonist ECR's MEP amplitudes were not. Previous studies on reciprocal muscles have shown that spinal disynaptic reciprocal inhibition is produced via activation of Ia inhibitory interneurons by Ia afferent input from the contracting agonist muscle (Crone, Hultborn, Jespersen, & Nielsen, 1987; Day, Marsden, Obeso, & Rothwell, 1984; Kagamihara & Tanaka, 1985; Katz, Penicaud, & Rossi, 1991; Tanaka, 1974). Moreover, the central nervous system also ensures that antagonist muscle activity is suppressed. This presumably facilitates Ia inhibitory interneurons in the corticospinal tract or inhibitory volleys that descend from the motor cortex to motor neurons in the antagonist muscle (Gerachshenko & Stinear, 2007; Giacobbe et al., 2011; Hoshiyama et al., 1996; Suzuki et al., 2012; Yang, Minn, Son, & Suk, 2006). In our study, we expected that corticospinal excitability changes would divergently affect agonist FCR and antagonist ECR muscles during behavioral tasks. However, negative outcomes affected agonist FCR MEP amplitudes only, and the performance or nonperformance of wrist flexion did not affect antagonist ECR MEP amplitudes. Although we cannot explain the mechanism by which unexpected penalties increased agonist FCR MEP amplitudes but not antagonist ECR MEP amplitudes, one possibility is that joint angular velocities were unrelated to reward probabilities. Although previous studies (Borgomaneri et al., 2012; Coelho et al., 2010; Oliveri et al., 2003) showed that corticospinal excitability was modulated by viewing negatively valenced pictures, we focused on muscle‐related corticospinal excitability changes associated with behavioral outcome expectations. Reward probabilities did not reflect the speed of wrist flexion and so may not have influenced joint angular velocity, which would weaken the relationship between reciprocal inhibitory function and reward probabilities. However, the role of changes in joint angular velocity and reciprocal inhibitory function is unclear in our study. Further research is therefore needed to investigate whether reciprocal inhibitory functions change in response to joint angular velocities in tasks with variable reward probabilities. Previous studies also noted that TMS‐evoked MEP amplitudes were depressed by fatigue (Gandevia, 2001; Milanović et al., 2011). This fatigue‐induced MEP depression is associated with central fatigue at corticospinal synapses (Gandevia, 2001; Gandevia, Allen, Butler, & Taylor, 1996). However, the time course of changes in FCR and ECR MEP amplitudes with and without wrist flexion were generally stable in our study. It is therefore unlikely that the changes in FCR MEP amplitudes were fatigue‐induced.

During reward processing, the M1 is influenced by many brain regions, including the ventral tegmental area, striatum, amygdala, and prefrontal cortex (Hikosaka, Bromberg‐Martin, Hong, & Matsumoto, 2008; Ikemoto, 2007; Schultz, 2004; Wickens et al., 2003). Neuronal activity in these areas increases or decreases in response to rewards or penalties, respectively (Schultz et al., 1997), and this is thought to improve behavioral choices by strengthening circuits associated with rewarded behaviors. Avoidance of nonrewarded behavior may reflect interactions between cortical and subcortical structures including the amygdala that process aversive stimuli (Borgomaneri et al., 2014; Oya, Kawasaki, Howard, & Adolphs, 2002; Tamietto & de Gelder, 2010). Moreover, previous studies have suggested that errors arising from rejection of a default option cause more regret than errors from acceptance do. Functional neuroimaging studies have also reported a critical role for the medial prefrontal cortex and insula in rejecting default options (Braver, Barch, Gray, Molfese, & Snyder, 2001; Camille et al., 2004; Carter et al., 1998; Chandrasekhar, Capra, Moore, Noussair, & Berns, 2008; Chua, Gonzalez, Taylor, Welsh, & Liberzon, 2009; Liu et al., 2007; Menon, Adleman, White, Glover, & Reiss, 2001; Nicolle et al., 2011). These areas may have been related to negative behavioral outcomes in our study. Such asymmetrical regret generation promotes status quo bias in subsequent decisions, especially difficult ones (Fleming et al., 2010; Nicolle et al., 2011; Pisoni et al., 2014). In trials where wrist flexion incurred penalties, agonist FCR MEP amplitudes increased. This might be related to regret associated with default option rejection. This regret about an unexpected penalty might have increased corticospinal excitability. Furthermore, Bestmann et al. (2008) noted that corticospinal excitability during preparation for behaviors increased in trials with low uncertainty about the necessary action and unsurprising events. Likewise, corticospinal excitability before a reward stimulus increased in trials with low reward probabilities (Suzuki et al., 2014). These results suggest that prebehavioral preparatory M1 activity is affected by the context under which the choices are made, with low uncertainty about the necessary action and a low reward probability increasing preparatory M1 activity. In our study, TMS was delivered 1 s after the reward or penalty stimulus because we focused on muscle‐related corticospinal excitability changes associated with positive and negative prediction errors. Although prebehavioral preparatory M1 activity and postbehavioral reward‐related M1 activity differentially affect MEP amplitudes, preparatory M1 excitability might be increased by low uncertainty and reward probability, whereas reward‐related M1 excitability might be increased by regret due to rejecting the default option. However, we did not perform any functional neuroimaging, so it is unclear whether M1 excitability is affected by activity in the amygdala, medial prefrontal cortex, and insula, which would be expected in the case of regret due to rejecting the default option. Further research should therefore be conducted into this question using both TMS and functional neuroimaging.

A potential limitation of this study is that using surface EMG carries a risk of recording crosstalk signals from nontarget muscles in close proximity to the target muscle. A previous study (Selvanayagam, Riek, & Carroll, 2012) using surface and fine‐wire EMGs noted similar correlations between FCR and ECR response amplitudes following radial or median nerve stimulation. Although crosstalk between agonist and antagonist muscles might be present in surface EMG, we found that negative outcomes increased agonist FCR MEP amplitudes but did not affect antagonist ECR MEP amplitudes, a difference that might exceed any present crosstalk. One possible explanation for the changes in agonist muscle MEP amplitudes might be that transmission efficiencies at agonist muscle‐related synapses might be increased in negative outcome‐activated brain regions including the striatum, amygdala, hippocampus, thalamus, prefrontal cortex, and insula. However, we could not directly observe such neuronal activity. Single‐unit recordings from dopaminergic neurons (Fiorillo, Tobler, & Schultz, 2003) suggest that reward and penalty outcomes are encoded in sustained firing. A study by Koepp et al. (1998) using ^11^C‐labeled raclopride and positron emission tomography scans found evidence that endogenous dopamine was released in the human striatum during a behavioral task. Another ^11^C‐labeled raclopride positron emission tomography study in which subjects performed monetary reward tasks noted that rewards increased dopamine transmission (Zald et al., 2004). More research should be performed into the neuronal effects of positive and negative outcomes using both TMS and brain imaging methods.

## 5 CONCLUSIONS

In conclusion, we found that greater reward probabilities were associated with more wrist flexions and that penalties increased corticospinal excitability for agonist muscles during a trial‐and‐error decision‐making task. In fact, corticospinal excitability for agonist muscles increased more in response to unexpected penalties than to expected rewards. These results imply that corticospinal excitability for agonist muscles can be differentially altered by positive and negative outcomes and might be specific to reward‐related behavioral selection. These findings have implications for motor learning and trial‐and‐error decision‐making, both of which partly rely on corticospinal excitability including the M1 and on learning from unexpected rewards and penalties. This study also provides further evidence that TMS provides a useful means of monitoring reward‐related corticospinal activity during trial‐and‐error decision‐making.

## CONFLICT OF INTEREST

The authors have declared that no conflicts of interest exist.
